# Controlled Release of Growth Factor from Heparin Embedded Poly(aldehyde guluronate) Hydrogels and Its Effect on Vascularization

**DOI:** 10.3390/gels9070589

**Published:** 2023-07-21

**Authors:** Yilan Zhao, Zezhong Lin, Wenqu Liu, Mingwei Piao, Junjie Li, Hong Zhang

**Affiliations:** 1 School of Chemical Engineering and Technology, Tianjin University, Tianjin 300072, China; 2 School of Science, Tianjin University, Tianjin 300072, China; 3 Key Laboratory of Resource Chemistry and Eco-Environmental Protection in Tibetan Plateau of State Ethnic Affairs Commission, School of Chemistry and Chemical Engineering, Qinghai Minzu University, Xining 810007, China; 4 School of Materials Science and Engineering, Tianjin University, Tianjin 300072, China; 5 Frontiers Science Center for Synthetic Biology and Key Laboratory of Systems Bioengineering (Ministry of Education), Tianjin University, Tianjin 300350, China

**Keywords:** alginate hydrogel, heparin, growth factor, vascularization, drug delivery

## Abstract

To deliver growth factors controllably for tissue regeneration, poly(aldehyde guluronate) (PAG) was obtained from alginate and covalently cross-linked with aminated gelatin (AG) to form PAG/AG hydrogel as a growth factors carrier. The prepared hydrogel exhibits a slow degradation rate and excellent cytocompatibility. Heparin was conjugated with gelatin and embedded into the hydrogel to reserve and stabilize growth factors. Basic fibroblast growth factor (bFGF) was immobilized into the hydrogel and performed sustained release as the hydrogel degraded. The bFGF loaded hydrogel can improve vascularization effectively in a rat dorsal sac model. To summarize, heparin embedded PAG/AG hydrogels would serve as a promising biodegradable vehicle for the controlled delivery of growth factors and promoting vascularization in regenerative medicine.

## 1. Introduction

Hydrogels, a class of cross-linked water-soluble polymer networks, have been widely used as cell scaffold or drug delivery vehicles in tissue engineering. Except for good biocompatibility and biodegradability, the ideal hydrogels would mimic certain advantageous properties of natural extracellular matrix (ECM) to provide a functional microenvironment for cell growth and drug releasing [1,2,3]. Growth factors are signaling peptides that stimulate cell activities and thus promote tissue repair and regeneration, which has been proved to be an important and routinely used drug in tissue engineering [4,5,6]. To date, the controllable and sustained delivery of growth factors remains a challenge. Many synthetic and naturally derived materials have been processed into hydrogels for growth factor delivery, and the binding affinity of hydrogels to growth factors and hydrogels degradation would play significant roles in release behaviors.

Alginate (ALG) is a linear block polyanionic natural copolymer, composed of 1,4-glycosidic linked D-mannuronic acid (M-units) and L-gluronic acid (G-units) residues. Its good biocompatibility, hydrophilicity, and low cost have made ALG an important candidate in biomaterials [7,8,9]. However, the weak mechanical stiffness and the degradation behavior of ALG with a relatively high content of M-units are always unsatisfactory due to the free rotation around the glycosidic linkages [10]. In our previous study, ALG scaffold containing disulfide bonds was prepared via cross-linking by a condensation of cystamine with diterminated amino groups, and reducible decomposition of hydrogels in physiological condition was achieved [11]. Although mechanically reinforced by surface coating of chitosan via electrostatic interactions, most alginate-based hydrogels degrade in a short time, which limits their applications in growth factor delivery [12,13,14]. In a seminal study by Mooney et al., M-units were removed from ALG to produce polyguluronate (PG) and oxidized to poly(aldehyde guluronate) (PAG) [15]. PAG was cross-linked with hydrazide derivatives to form hydrogels, which exhibit a wide range of stiffness and controllable degradation rates [16,17]. Moreover, PAG hydrogels have demonstrated their potential for both the release of antineoplaston in vitro and performing as cell scaffolds in bone tissue engineering [18,19].

Growth factors can promote cell growth and differentiation, playing an important role in wound healing and organ repairing in situ, hence growth factors have been widely used in tissue engineering and regenerative medicine [20,21,22]. However, the application of growth factors is limited because of their burst release and poor stability in vivo [23,24]. It is necessary to construct a delivery system to realize the long-term controlled release of growth factors in situ. Heparin, a major family of highly sulfated polysaccharide in glycosaminoglycans (GAGs), has been shown to interact with diverse proteins. Many growth factors directly bind to the ECM by interacting with the heparin component in GAGs, so that the ECM is able to protect growth factors from degradation and regulate the distribution of growth factors in tissue [25,26,27,28]. Therefore, heparin has been used as a binder and stabilizer in the delivery of growth factors [29].

In this study, we removed the M-units from ALG according to the protocol reported by Mooney et al. [15], which was cross-linked with aminated gelatin (AG) to form PAG/AG hydrogels. Hydrogels exhibiting a significantly slower and better controllable degradation rate are anticipated to function as effective carriers for the delivery of growth factors. In addition, as a strategy to protect and stabilize growth factors in hydrogels, heparin and gelatin were conjugated (HGC) and embedded in hydrogels to mimic the structure of GAGs. Basic fibroblast growth factor (bFGF) was chosen as a model to be immobilized on HGC and loaded in hydrogels. The cytotoxicity and degradation of PAG/AG hydrogels, the release of bFGF from hydrogels, and the vascularization of bFGF loaded hydrogels in vivo were investigated.

## 2. Results and Discussion

### 2.1. Preparation of Cross-Linked PAG/AG Hydrogels

**Figure 1:**
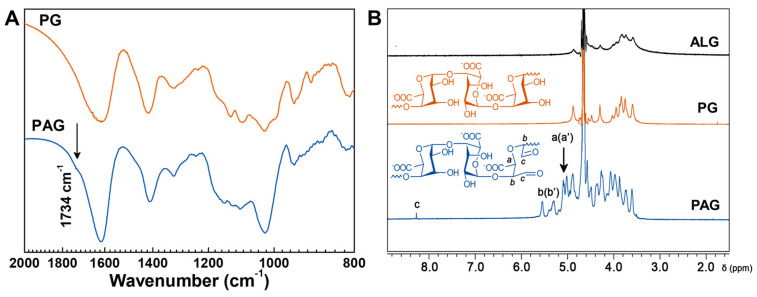
Characterization of PAG. (**A**) FTIR spectra of PA and PAG; (**B**) ^1^H NMR spectra of ALG, PG, and PAG in D_2_O. (**B**) Chemical structures of PG and PAG.

In this study, PG was isolated from ALG by acid hydrolysis to remove M-units. Next, the vicinal diols in PG were cleaved by sodium periodate (NaIO_4_) to form PAG with aldehyde groups. The FTIR spectra of PG and PAG are shown in Figure 1A. Compared to PG, PAG appears with a new peak at 1734 cm^−1^, which corresponds to the aldehyde stretching vibrations. The formation of PAG was further investigated by ^1^H NMR, as shown in Figure 1B. The chemical shifts at 5.01 and 5.09 ppm (a, a′) belong to protons on C-5, and signals at 5.30 and 5.55 ppm (b, b′) attribute to protons on C-1 and C-4. The characteristic proton peak of aldehyde groups (oxidized position on C-2 and C-3) appears at 8.28 ppm (c) [30,31]. FTIR and ^1^H NMR analysis both show that aldehyde groups have been introduced in PAG using our method.

**Figure 2:**
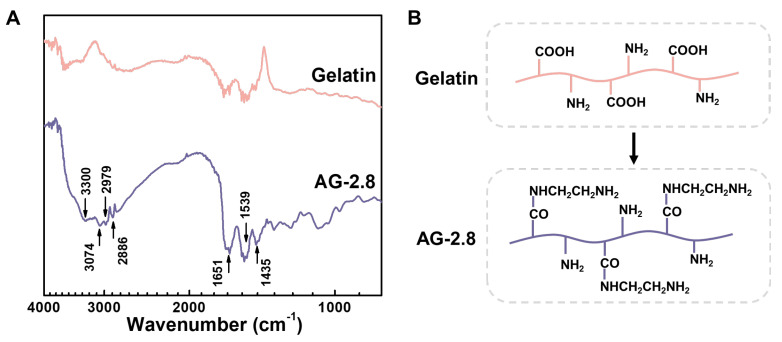
(**A**) FTIR spectra of gelatin and AG-2.8, and (**B**) their chemical structures.

The hydrogels were cross-linked through Schiff’s reaction between aldehyde groups in PAG and amino groups in gelatin. Type A gelatin was chosen herein for its relatively high nitrogen content, but the original amino groups were still limited. The carboxyl groups of gelatin were thus modified with ethylenediamine (EDA) to increase its content of amino groups. The FTIR spectra of free gelatin and AG are shown in Figure 2A. After modification, peak intensities at 3300, 3074 (N-H stretching vibration) and 1539 cm^−1^ (N-H bending vibration) are increased. The peaks at 1651 and 1435 cm^−1^ correspond to C=O stretching vibration and C-N stretching vibration, respectively, which appears for the coupling reaction. In addition, peaks at 2979 and 2886 cm^−1^ of stretching vibration of methylene arising from EDA are enhanced compared to that of free gelatin. By changing the feed ratio of EDA to gelatin, the modified gelatin is controlled. Using 2,4,6-trinitrobenzenesulfonic acid (TNBS) assay, the amino groups in AG are quantified, as listed in Table 1. It is found that the content of amino groups increases with increasing the amount of EDA, achieves a maximum of ca. 0.8 mmol g^−1^, and keeps relatively constant when the feed ratio reaches 2.8. In a previous study, Ikaka and co-workers modified the carboxyl groups of gelatin into amino groups similarly using EDA. They verified that the amount of amino groups that can be introduced reaches a maximum until the [EDA]/[COOH] ratio reached 20, with no further increase at [EDA]/[COOH] ratios above 20. Our results agree strongly with the trend in Ikada’s study, which implies there may exist saturated carboxyl groups of gelatin when the feed ratio reaches a certain value. The excess EDA cannot graft to gelatin but is removed through dialysis, which is consistent with the previous study [32]. Therefore, we chose a feed ratio of 2.8 as the optimum condition and AG-2.8 was used in this study.

**Figure 3:**
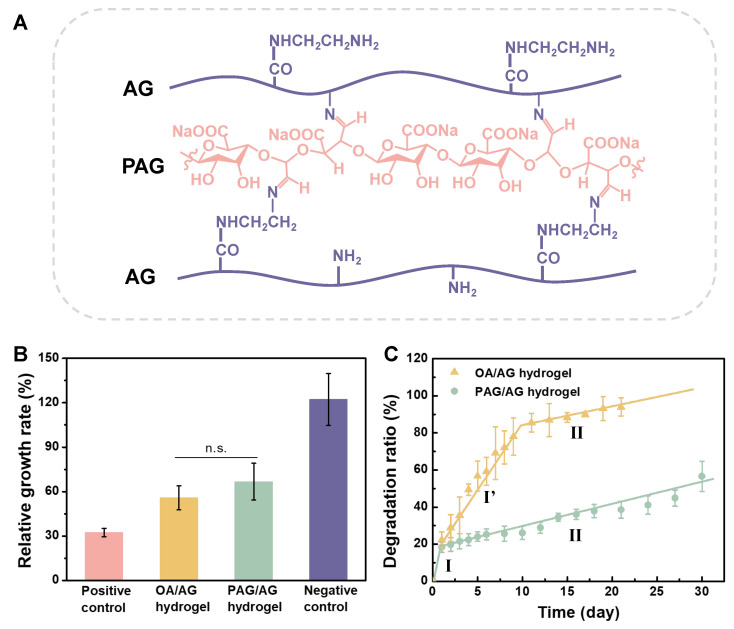
(**A**) Chemical structure of crosslinked PAG/AG hydrogels. Cytotoxicity and degradation of PAG/AG and OA/AG hydrogels in vitro. (**B**) Relative growth rate of L929 cell proliferation in the extraction medium from hydrogels. (**C**) degradation ratio of hydrogels in PBS. (n.s.: no significant difference).

After mixing PAG and AG at 37 °C for 24 h, cross-linked PAG/AG hydrogels were formed. The chemical structure of the PAG/AG hydrogels is illustrated in Figure 3A. Prior to the application in vivo, cytotoxicity and degradation behavior of the hydrogels should be taken into consideration. We investigated the cell proliferation in the extraction medium from the PAG/AG hydrogels through MTT assay. Oxidized alginate/aminated gelatin (OA/AG) hydrogels were studied as well. Relative growth rate of PAG/AG and OA/AG is 66.8 ± 12.4% and 55.8 ± 8.1%, respectively, as shown in Figure 3B. Statistical analysis reveals that the difference between PAG/AG and OA/AG is insignificant. Therefore, we consider that both of the two hydrogels performed good cytocompatibility according to United States Pharmacopeia [33], and the changed component from OA to PAG had no significant effect on the cytocompatibility of the hydrogels.

The degradation ratio of PAG/AG and OA/AG hydrogels were investigated in physiological conditions, as shown in Figure 3C. For PAG/AG hydrogels, a dramatic weight loss of ca. 20% occurred in the first day (Stage I). From Day 2, degradation exhibited a uniform slow rate, and ca. 55% of hydrogels were still preserved after one month (Stage II). For OA/AG hydrogels, however, degradation can be divided into three stages: fast degradation in the first day (Stage I), medium rate degradation from Day 2 to Day 10 (Stage I′), and slow degradation from Day 11 (Stage II). The OA/AG hydrogels were almost completely degraded after 21 days. In this study, an excess amount of AG was used to improve the biocompatibility of hydrogels (mass ratio of PAG or OA to AG is 1:6); therefore, the degradation in Stage I can be attributed to the burst dissolution of unreacted AG. In Stage II, the cleavage of C=N in Schiff’s structure in cross-linked hydrogels is deemed to be a dominant factor for degradation. The networks formed from OA/AG are fragile due to the existence of M-units. We believe that the degradation in Stage I′ results from a combined mechanism involving hydrolysis of OA chains and cleavage of C=N bonds simultaneously. According to the previous reports, during the cleavage of C=N bonds, aldehyde groups on PAG can be revived and re-cross-linked before the complete hydrolysis of PAG chains, which decelerates the degradation of PAG/AG hydrogels [16,17]. Taken together, we confirmed that by acid hydrolysis and oxidation from alginate, degradation of cross-linked PAG/AG hydrogels is steady and moderate compared to that of OA/AG hydrogels, which may provide a promising degradable scaffold for release of growth factor in vivo.

### 2.2. Release of bFGF from PAG/AG Hydrogels In Vitro

HGC was prepared by reductive amination, where the hemiacetal carbon of heparin was attacked by lone pair electrons on amino groups of gelatin. TB assay was applied to determine that the amount of heparin in HGC is ca. 48.3%. Taking into account the molecular weights of heparin (ca. 12 kDa) and gelatin (ca. 100 kDa), it is estimated that HGC contains around eight heparin molecules per each gelatin backbone. Previous studies have clarified that *O*-sulfate groups and *N*-sulfated glucosamines in heparin are essential for binding of bFGF [26]. In this study, the binding affinity of HGC to bFGF is anticipated to be comparable to that of free heparin, as there is no chemical modification of the functional groups as mentioned above.

**Figure 4:**
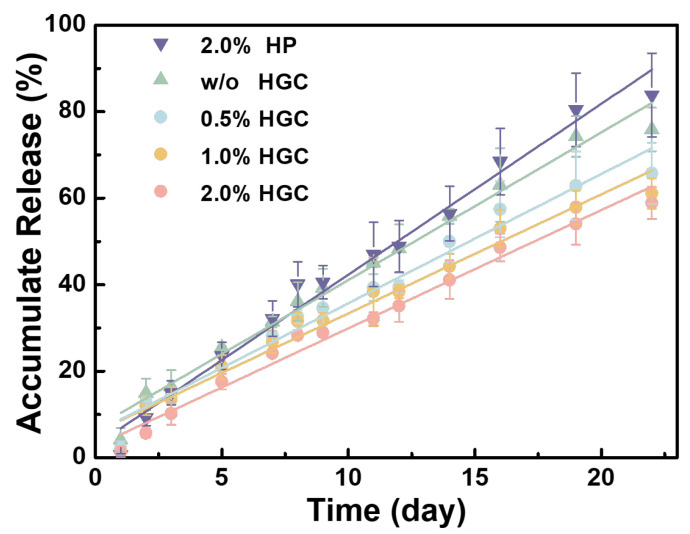
Accumulate release (fitted curves) of bFGF from PAG/AG hydrogels within three weeks.

bFGF was immobilized on HGC by incubation, which was subsequently loaded into PAG/AG hydrogels. Due to the abundant amino groups in gelatin, bFGF immobilized HGC participates in the formation of PAG/AG hydrogels. bFGF is quantified by enzyme-linked immunosorbent assay (ELISA) and its release behavior from hydrogels was traced within three weeks, as shown in Figure 4. The absolute load amount of bFGF in 0.5 cm^3^ of PAG/AG hydrogels was fixed to 2.0 μg, and the weight ratio of heparin component in HGC to the hydrogels was adjusted to 0.5%, 1.0%, and 2.0%, referred as 0.5% HGC, 1.0% HGC, and 2.0% HGC, respectively. Samples with 2.0% heparin (without gelatin) and those without HGC or heparin (direct bFGF loading) were studied as well, referred to as 2.0% HP and w/o HGC, respectively. Results show that PAG/AG hydrogels can sustainably release bFGF within 21 days without burst release in all cases. The amount of heparin in hydrogels was found significant in determining the rate of bFGF release. For instance, if we consider the result on Day 16, the accumulate release from 0.5% HGC, 1.0% HGC, and 2.0% HGC are 57.4%, 53.0%, and 48.6%, while on Day 21 such percentages become 65.7%, 61.1%, and 58.9%, respectively. It is found the rate of bFGF release decreases with increasing the amount of heparin in hydrogels, in agreement with a previous study [34,35]. However, the release behaviors of 2.0% HP and 2.0% HGC exhibit notable differences, although the amounts of heparin in these two hydrogels are the same. The accumulated release from 2.0% HP and 2.0% HGC are similar to each other in the beginning of release, which become 68.5% and 48.6% on Day 16, and further extend to 83.8% and 58.9% on Day 21, respectively. The bFGF release from 2.0% HP is comparably fast or even faster than that from w/o HGC, i.e., bFGF-loaded PAG/AG hydrogels without HGC or heparin.

**Figure 5:**
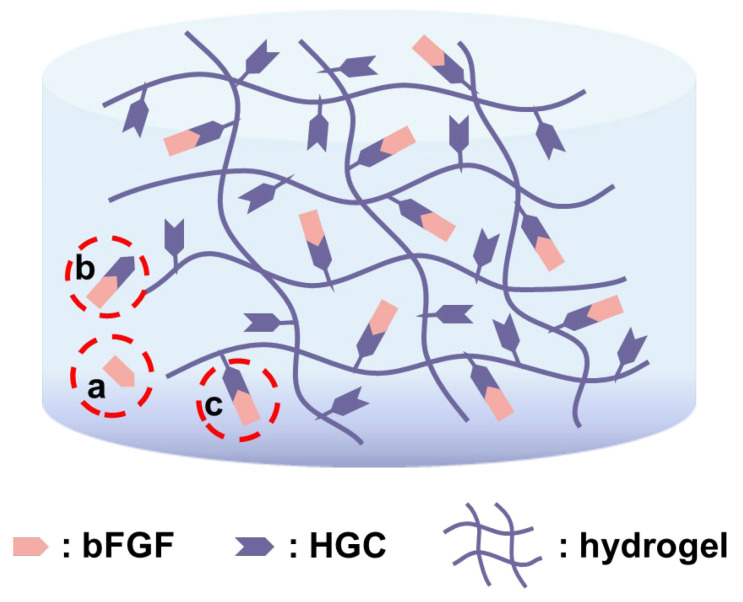
Schematic representation of three possible forms of bFGF that exists in hydrogels.

There are mainly two mechanisms for drug release in hydrogels [36]. One is degradation/erosion control, where drug molecules are released in tandem with the disaggregation of hydrogels. The other mechanism is diffusion control, where water/medium diffuses into a swollen hydrogel to dissolve the loaded drug molecules. Here, three forms of bFGF may exist in the hydrogels, i.e., free bFGF (a), free HGC-immobilized bFGF (b), and covalently bonded HGC-immobilized bFGF (c), as shown in Figure 5. Heparin and gelatin were conjugated through a strong and stable C-N bond, and thus heparin-bFGF complex does not appear during the release process. In this experiment, HGC was thoroughly reacted with PAG and then an excess amount of AG was added, which means that all the HGC can be linked to PAG/AG hydrogels. Thus, bFGF releasing with HGC is attributed to the hydrogel degradation, i.e., the cleavage of C=N in Schiff’s structure, as discussed above. The degradation of hydrogels and the release of bFGF exhibit a similar linear behavior, also suggesting their correlations. With increasing the heparin in hydrogels, more bFGF is immobilized and less bFGF can be released in the form of free bFGF, which decelerates its release. In the case of 2.0% HP, however, free HGC-immobilized bFGF is physically loaded in hydrogels, and release is prone to be driven by a fast diffusion control. In addition, bFGF without HGC or heparin (w/o HGC), as a polypeptide, may be involved in cross-linking reactions of hydrogels and its releasing is controlled by hydrogel degradation. It should be noted that increasing the amount of heparin in hydrogels can provide a sustained release of bFGF for a long period; however, an overdose of heparin may lead to excessive bleeding. In the following in vivo study, we investigated the vascularization of bFGF loaded hydrogels with various ratios of HGC and 1.0% HGC hydrogels with various bFGF loadings.

### 2.3. Vascularization of bFGF Loaded PAG/AG Hydrogels In Vivo

The results of angiogenic response to the bFGF loaded hydrogels need to be evaluated, and the bioactivity of the released bFGF from hydrogels should be verified. It has been well known that bFGF can enhance the vascularization for bFGF induces endothelial cells to divide, migrate, and differentiate into tubular structures [37,38]. Herein, we studied the vascularization of bFGF loaded PAG/AG hydrogels with a rat dorsal sac model. Unlike other methods for evaluation of vascularization, such as a chick chorioallantoic membrane (CAM) model, the subfascial space of a rat model provides a rare vascular environment for hydrogel implantation, and all the observed vessels in hydrogels can be deemed as neovascular development, but not some preexisting vessels [39]. Since vascular growth is generally limited to several tenths of micrometers per day, one week was considered to be the minimum period for inducing sufficient vessels in a height of 2 mm hydrogel sample [40,41].

**Figure 6:**
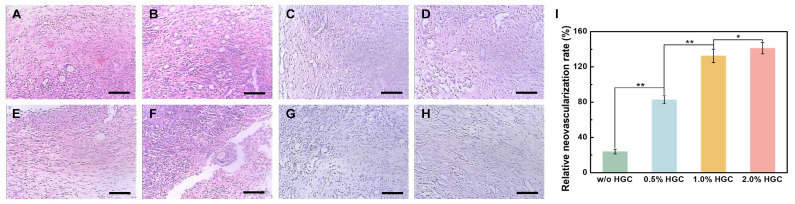
(**H**,**E**) Staining image of newly formed vessels in hydrogels with and without bFGF load after one week for samples: (**A**,**E**) w/o HGC, (**B**,**F**) 0.5% HGC, (**C**,**G**) 1.0% HGC, and (**D**,**H**) 2.0% HGC. Scale bar: 100 μm. (**I**) Relative neovascularization rate of PAG/AG hydrogels (* *p* < 0.05, ** *p* < 0.01).

To evaluate the angiogenic response to hydrogels with various ratios of HGC, the weight ratio of the heparin component in HGC to the implanted hydrogels was adjusted to 0% (without HGC), 0.5%, 1.0%, and 2.0%, referred as w/o HGC, 0.5% HGC, 1.0% HGC, and 2.0% HGC, respectively. The amount of bFGF loaded in the hydrogels was fixed to 100 ng mL^−1^. Hydrogels without bFGF were implanted as controls. As shown in Figure 6, after implantation, a few new vessels were observed in control groups, and the inflammatory reaction mainly consisted of necrotic neutrophils (Figure 6E–H). For bFGF loaded samples, a small amount of new vessels generated in w/o HGC and 0.5% HGC groups, infiltrated mainly by lymphocytes and monocytes (Figure 6A,B), while more new vessels were formed in 1.0% HGC and 2.0% HGC groups and showed chronic inflammatory cell infiltration (Figure 6C,D). The results were consistent with previous research [42,43]. The neovascular number can be quantified by imaging analysis [44,45]. The relative neovascularization rates of w/o HGC, 0.5% HGC, 1.0% HGC, and 2.0% HGC groups were calculated to be 24.00%, 82.79%, 132.5%, and 141.3%, indicating that hydrogels having more HGC improved the efficacy of bFGF for promoting neovascularization more effectively. This is because the heparin component in HGC can protect bFGF and extended its half-life [28,29].

**Figure 7:**
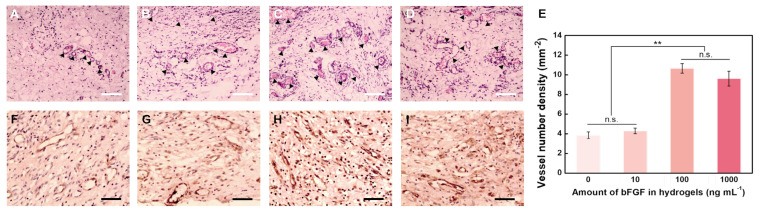
H & E staining image of newly formed vessels in hydrogels after one week with loading amount of bFGF of (**A**) 0, (**B**) 10, (**C**) 100, and (**D**) 1000 ng mL^−1^ (arrows show newly formed vessels). Scale bar: 100 μm. (**E**) Vessel number densities in hydrogels with different loading amounts of bFGF (n.s.: no significant difference, ** *p* < 0.01). Immunohistochemical staining for Factor VIII-related antigen images of newly formed vessels in hydrogels after one week with loading amount of bFGF of (**F**) 0, (**G**) 10, (**H**) 100, and (**I**) 1000 ng mL^−1^. Scale bar: 50 μm.

To the verify the bioactivity of the released bFGF from hydrogels, the amount of bFGF loaded in hydrogels (1.0% HGC) was adjusted to 0, 10, 100, and 1000 ng mL^−1^. As shown in Figure 7A–D, after implantation, a certain number of vessels can be found in hydrogel treated areas (marked by arrows), while the tissue has some degree of edema. Without bFGF loading, the newly formed vessels exhibited capillary-like, and infiltrated by lymphocytes, neutrophilic granulocyte, and plasmocytes to the limited extent in Figure 7A,B. With bFGF and increasing its loading amount, better vascularized regions were formed in Figure 7C,D with more vessels, more vascular tortuosity, and thicker vascular wall, which was in agreement with previous reports [46,47]. The density of fibroblasts in hydrogels also increased with increasing bFGF loading amount, which is due to the effect of bFGF in promoting cell division and proliferation. The vessel number densities of PAG/AG hydrogels with 0, 10, 100, and 1000 ng mL^−1^ of bFGF loading were measured to be 3.85, 4.29, 10.65, and 9.62 mm^−2^, respectively (Figure 7E). Statistical analysis reveals that both the difference between 0 and 10 ng mL^−1^ and the difference between 100 and 1000 ng mL^−1^ bFGF are insignificant. However, there is a significant difference between these two groups.

Furthermore, immunohistochemical staining for Factor VIII-related antigen visualized the vascular endothelial cells in the vascular wall, as shown in Figure 7F–I. Although some lymphocytes and fibroblasts exhibit positive staining, it is clear that the group of hydrogels with 100 ng/mL bFGF loading is the most vascularized, in strong agreement with the above results from H and E staining. Therefore, we conclude that the released bFGF from the PAG/AG hydrogels can preserve its bioactivity and improve the vascularization in vivo, whose effect is controlled by the concentration of bFGF. For 1.0% HGC hydrogels, the relative neovascularization rate with a loading amount of 100 ng mL^−1^ is approximately 132.5%, which is similar to the efficacy of bFGF loaded materials reported previously by Garbern et al. (130%) and Jeon et al. (125.5%) [48,49]. The vascular growth cannot be enhanced furthermore by increasing the amount of bFGF above 100 ng mL^−1^, which is because of a limitation arising from the rate of bFGF release during the hydrogel degradation.

## 3. Conclusions

The weak stiffness and unsatisfactory degradation of alginate hydrogels always limits their usefulness, such as for growth factor delivery. To address this problem, we prepared poly(aldehyde guluronate) from alginate, which was covalently cross-linked with aminated gelatin. Results show that the novel hydrogels exhibit excellent cytocompatibility, and a much slower degradation rate within one month. Basic fibroblast growth factor is immobilized in hydrogels through conjugated heparin and gelatin to mimic the structure of GAGs. The binding affinity of heparin to bFGF is confirmed. The release of bFGF is attributed to hydrogel degradation, and the rate of release decreases with increasing the amount of heparin in hydrogels. Regarding the in vivo test, the released bFGF is enabled to preserve its bioactivity to improve vascularization in a rat dorsal sac model. For 1.0% heparin-gelatin conjugate embedded hydrogels, a loading amount of 100 ng mL^−1^ bFGF is found sufficiently efficacious. Although this work is still at an early stage, we anticipate it will be of practical value in the fabrication of biodegradable hydrogels as biomimetic vehicles for controllable drug delivery in tissue engineering.

## 4. Materials and Methods

### 4.1. Materials

Sodium alginate with a high content of guluronic acid of about 70% and a vis-cosity-average molecular weight of 540 kDa was received from FMC BioPolymer (Drammen, Norway). 1-ethyl-3(3-dimethylaminopropyl) carbodiimide hydrochloride (EDC·HCl) was purchased from GL Biochem (Shanghai, China). Gelatin (Type A), MTT assay kit and peroxidase-conjugated goat anti-rabbit IgG were obtained from Sigma-Aldrich Chemical Co. (Shanghai, China). Heparin was purchased from Huixing Biochemistry Reagent Co., Ltd. (Shanghai, China). Basic fibroblast growth factor (bFGF) was purchased from Pepro Tech Inc. (Suzhou, China). Bovine serum albumin (BSA, electrophoretic purity) was purchased from Chinese Academy of Medical Sciences Blood Research Institute Science and Technology Co., Ltd. (Tianjin, China) Penicillin/streptomycin was purchased from Harbin Pharmaceutical Group Holding Co., Ltd. (Harbin, China) Rabbit anti-human bFGF was purchased from AbD Serotec Ltd. (Oxford, UK). Specific pathogen free (SPF) rats (ca. 200 g) were purchased from Merial Vital Laboratory Animal Technology Co., Ltd. (Beijing, China). Mouse IHC kit was obtained from 4A Biotech Co., Ltd. (Beijing, China). Factor VIII antibody was purchased from Boster Biotechnology Co., Ltd. (Wuhan, China).

### 4.2. Preparation of Cross-Linked PAG/AG Hydrogels

#### 4.2.1. Synthesis of PAG

PAG was obtained from alginate (ALG) by the acid hydrolysis and oxidation procedure reported by Mooney et al. [15]. Herein, sodium alginate with a high content of guluronic acid of about 70% and a viscosity-average molecular weight of 540 kDa was used. 15 g of ALG was dissolved in 0.7 L of deionized water. 75 mL of 3 mol L^−1^ hydrochloric acid was added and the mixture was refluxed at 70 °C for 5 h. The solution was allowed to stand overnight and the precipitate was collected by centrifugation and suspended in 1 L of water. Next, 5.85 g of sodium chloride was added, followed by the addition of 10 mL of 4 mol L^−1^ sodium hydroxide. The pH was adjusted to 2.25 with 12 mol L^−1^ hydrochloric acid. The suspension was centrifuged and the white precipitate was washed with 100 mL of water and re-suspended again. Subsequently, 0.6 g of sodium chloride was added and the pH was adjusted to 7.5 with sodium hydroxide, followed by adding 4 g of activated carbon. The suspension was stirred for 2 h, centrifuged to remove activated carbon, and then the product of PG can be obtained after freeze-drying. PG was oxidized by sodium periodate (NaIO_4_) to yield PAG. Specifically, 10 g of PG and 50 mL of 0.5 mol L^−1^ NaIO_4_ aqueous solution were added in 100 mL of water and the reaction was stirred at 4 °C for 19 h. Ethylene glycol was added to stop the oxidation. The solution was precipitated and filtered by ethanol. The precipitate was collected, dissolved in water, and dialyzed (*M_W_* cutoff: 3500 Da) for 3 days. The solution was then freeze dried to yield the white product of PAG.

#### 4.2.2. Synthesis of AG

In typical, 10 g of gelatin and a certain amount of ethylenediamine (EDA) were dissolved in 250 mL of phosphate buffer saline (PBS, pH = 5.0) and pH was adjusted to 5.0 with hydrochloric acid. Subsequently, 5.35 g of EDC·HCl was added, which was adjusted to 500 mL with PBS and stirred at 37 °C for 1 h. The solution was dialyzed against water for 3 days and AG was obtained after lyophilization. The degree of modification on gelatin was evaluated by measuring the content of amino groups in AG. Specifically, 1 mL of 0.5 mg mL^−1^ AG in PBS (pH = 7.4) was mixed with 1 mL of 4% (*w*/*v*) sodium bicarbonate (NaHCO_3_) and 1 mL of 0.1% 2,4,6-trinitrobenzenesulfonic acid (TNBS) solution. The mixture was incubated at 40 °C for 2 h in the dark and the absorbance was measured at 415 nm using a Bio-Tek Synergy HT microplate reader. The standard solutions were prepared from β-alanine [50]. The content of amino groups in AG can be calculated by comparison with standard results. As to the characterization of chemical structure of PAG and AG, ^1^H NMR and Fourier transform infrared (FTIR) spectroscopy were applied. For ^1^H NMR, about 20 mg of ALG, PG, and PAG were dissolved in 0.5 mL of D_2_O, and spectra were acquired with an INOVA 500 MHz spectrometer (Varian Associates, Palo Alto, USA). The FTIR spectra of PG, PAG, free gelatin, and AG were recorded with a Bio-Rad FTS-6000 FTIR spectrometer.

#### 4.2.3. Fabrication of PAG/AG Hydrogels

The aldehyde groups of PAG and the amino groups of AG can be covalently cross-linked through Schiff’s reaction. In brief, 5% (*w*/*v*) PAG and 30% (*w*/*v*) AG in PBS (pH = 7.4) was incubated at 37 °C for 30 min after complete dissolution. These two solutions were mixed at 1:1 ratio (*v*/*v*) in six-well tissue culture plates and incubated at 37 °C for 24 h to form cross-linked PAG/AG hydrogels.

#### 4.2.4. Cytotoxicity of PAG/AG and OA/AG Hydrogels

The cytotoxicity of PAG/AG and OA/AG hydrogels was evaluated by MTT assay. L929 fibroblast cells were seeded at 1 × 10^4^ cells on a 96-well tissue culture plate in 100 μL medium and incubated in a 5% CO_2_ at 37 °C for 24 h. The PAG/AG and OA/AG hydrogels were subjected to ^60^Co radiation sterilization and immersed in fresh RPMI 1640 medium at a 1:10 ratio (*v*/*v*) without cells at 37 °C for 24 h to accumulate any possible leach-out product. The medium from each well containing the cells was removed and replaced with 100 μL of extraction medium containing leach-out extract from PAG/AG hydrogels or OA/AG hydrogels, with culture medium as blank, 5 g L^−1^ phenol solution in culture medium as positive control, and extraction medium from sterilized high-density polyethylene (HDPE) as negative control. After L929 fibroblast cells were cultured in the extracts for 72 h, 20 µL of 5 g L^−1^ 3-(4,5-dimethylthiazol-2-yl)-2,5-diphenyltetrazolium bromide (MTT, Sigma-Aldrich) was added in each well and continued the culture for 4 h. Next, the suspension was removed and replaced with 100 μL of dimethyl sulfoxide (DMSO) for 10 min. The absorbance was measured at 570 nm using a microplate reader. The relative growth rate was calculated as *A*/*A*_0_ × 100%, where *A* presents the absorbance of samples or positive/negative control, and *A*_0_ presents the absorbance of blank medium [51].

### 4.3. Degradation of PAG/AG Hydrogels

The degradation behavior of PAG/AG hydrogels was investigated by a gravimetric method. The hydrogels were cut into a cylinder shape with ca. 0.5 cm^3^, and the lyophilized samples were first weighed (initial weight, *W_0_*). Next, the hydrogels were soaked in 2.5 mL of PBS (pH = 7.4) at 37 °C. At predetermined time intervals of one day, the sample was removed from PBS, wiped dry with filter paper on the surface, lyophilized, and weighted (*W_d_*). For other samples, 1.5 mL of PBS was withdrawn and replaced with fresh PBS. The degradation ratio was calculated using Equation (1):

Degradation ratio = (*W*_0_ − *W_d_*)/*W*_0_ × 100% (1)

The experimental results were obtained from quintuplicate samples. The degradation of cross-linked oxidized alginate/aminated gelatin (OA/AG) hydrogels was investigated as well. ALG was directly oxidized by NaIO_4_ to yield OA, without prior acid hydrolysis. Fabrication of OA/AG hydrogels and study of their degradation were conducted using the same method as described above.

### 4.4. Synthesis of HGC

HGC was synthesized by reductive amination. Briefly, 272 mg of heparin and 14.2 mg of gelatin were dissolved in 12 mL PBS (pH = 8.0). Next, 9.96 mg of sodium cyanoborohydride (NaBH_3_CN) was added and reacted at 40 °C for 24 h. Isolation of HGC was carried out with adding 1.5-time volume 95% (*v*/*v*) alcohol saturated with sodium acetate, and gentle stirred at room temperature for 30 min and centrifuged. The precipitate was re-dissolved in 12 mL PBS containing 4 mol L^−1^ guanidine hydrochloride, which was then dialyzed (*M*_W_ cutoff: 14,000 Da) for 3 days and HGC was obtained after lyophilization. The amount of heparin in HGC was determined by Toluidine blue (TB) assay [52]. Specifically, HGC solution (1 mg mL^−1^) and a series of heparin standard solutions (0–20 µg mL^−1^) were prepared in PBS (pH = 8.0). Subsequently, 1.0 mL of 0.0005% TB solution and either 0.1 mL of HGC solution or heparin standard were mixed, and 1 mL hexane was added and the mixture was separated into two layers. The aqueous layer was transferred into a 96-well tissue culture plate and the absorbance was measured at 631 nm using a microplate reader. Heparin content in HGC can be calculated by comparison with standard results.

### 4.5. Release of HGC-Immobilized bFGF from PAG/AG Hydrogels

bFGF was immobilized by dissolving HGC in 0.2 mL of 10 μg mL^−1^ bFGF solution at 37 °C for 1 h. The weight ratio of heparin component in HGC to the result hydrogels was adjusted to 0.5%, 1%, and 2%. Samples without HGC and those with 2% heparin were prepared as controls. The above solutions were firstly mixed with 0.8 mL of 8.3% (*w*/*v*) PAG, and then 1.0 mL of 30% (*w*/*v*) AG in PBS (pH = 7.4), which was incubated at 37 °C for 24 h to form HGC-immobilized bFGF loaded PAG/AG hydrogels. The hydrogels were cut into a cylinder shape with ca. 0.5 cm^3^ and immersed in 2.5 mL of PBS (pH = 7.4) containing 10 mg mL^−1^ bovine serum albumin (BSA), 10 µg mL^−1^ heparin, and 1000 U mL^−1^ penicillin/streptomycin at 37 °C. At predetermined time intervals of one day, 2.0 mL bFGF release medium was withdrawn and replaced with fresh PBS. The amount of bFGF was determined by a double-antibody sandwich enzyme-linked immunosorbent assay (ELISA) [44,53]. Briefly, a series of 100 μL of bFGF standard solutions (0.05, 0.10, 0.15, 0.20, and 0.25 μg mL^−1^) were added in a 96-well ELISA plate and incubated at 37 °C for 2 h. The plate was then washed three times with PBS (pH = 7.4) containing 0.05% (*v*/*v*) Tween-20. Next, 100 μL of rabbit anti-human bFGF at 1:10,000 dilution was added, incubated for 1 h. and washed three times with PBS. Afterwards, 100 μL of peroxidase-conjugated goat anti-rabbit IgG at 1:2000 dilution was added, incubated for another 1 h, and washed three times with PBS. Finally, 100 μL of 0.1 mol L^−1^ citrate buffer (pH = 5.0) containing 0.4 mg mL^−1^ 1,2-phenylenediamine and 0.03% (*v*/*v*) hydrogen peroxide was added and allowed to stand for 15 min in the dark. The reaction was stopped by the addition of 50 μL 1 mol L^−1^ sulfuric acid. The absorbance was measured at 490 nm. 100 μL of bFGF release mediums were conducted using the same method and the concentration of bFGF in the release medium can be obtained by comparison with standard results. The accumulated release of bFGF from hydrogels (*E_r_*) was calculated using Equation (2):

*E_r_* = [*V_e_* (*C*_1_ + *C*_2_ + ··· + *C_n-_*_1_) + *V*_0_*C_n_*]/*m_f_* × 100%, (2)

where *V_e_* and *V*_0_ represent the volume of replacement PBS (2.0 mL) and total release medium (2.5 mL), *C_i_* and *C_n_* is the concentration of bFGF in the release medium after *i* and *n* times replaced the PBS, and *m_f_* is the original loading amount of bFGF in PAG/AG hydrogels (2.0 μg).

### 4.6. Vascularization of bFGF Loaded PAG/AG Hydrogels In Vivo

#### 4.6.1. Preparation of Rat Dorsal Sac Model

The bFGF loaded hydrogels were prepared as described above. The weight ratio of heparin component in HGC to the prepared hydrogels was adjusted to 0% (without HGC), 0.5%, 1%, and 2%. Next, the hydrogels were cut into a cylinder shape with a diameter of ca. 5 mm and a height of ca. 2 mm and subjected to ^60^Co radiation sterilization before animal experiments. Specific pathogen free (SPF) rats (ca. 200 g) were anesthetized by intraperitoneal injection of ketamine, and seven rats were used in each group (n = 7). Four ca. 6 mm incisions were horizontally made in the subcutaneous back region. Hydrogels were then implanted into the dorsal sac region and sutured. Hydrogels without bFGF were implanted into SPF rats as controls. After one week, the rats were euthanized, and the tissues surrounding the hydrogels were carefully dissected [54]. Samples were fixed with 4% formalin and dehydrated in a series of ascending concentrations of ethanol. The paraffin-embedded sections (5 μm) were prepared (Leica EG 1150H and RM 2255). Animal experiments were conducted according to the National Institutes of Health (NIH) Guide for Care and Use of Laboratory Animals, and the surgical procedures were approved by the Animal Ethics Committee of Tianjin University.

#### 4.6.2. Histological Observation

The sections were stained with hematoxylin and eosin (H and E) with standard protocols. Immunohistochemical staining for Factor VIII-related antigen was performed as well. Briefly, the sections were treated with 3 wt% hydrogen peroxide for 10 min and blocked with 5 wt% goat serum (from Mouse IHC kit) at 37 °C for 30 min. Sections were incubated with Factor VIII antibody (1:200) at 4 °C overnight and then washed with PBS followed by incubation with biotinylated goat anti-rabbit secondary antibody at 37 °C for 20 min. After being washed with distilled water, the sections were treated with streptavidin-biotin complex and 3,3′-diaminobenzidine. All the sections were observed under an upright microscope (Olympus BX50). The total vessel number was quantified by ImageJ software. Vessel number density was calculated using Equation (3):

Vessel number density = *N*/*A* × 100%, (3)

where *N* represents the vessel number counted by ImageJ, and *A* is the area analyzed by ImageJ during count. Relative neovascularization rate was calculated using Equation (4):

Relative neovascularization rate = (*D_e_* − *D_c_)*/*D_c_* × 100%, (4)

where *D_e_* and *D_c_* represent the vessel number density of experimental groups and controls.

### 4.7. Statistical Analysis

All quantitative data were expressed as the means ± s.d. (n ≥ 5). Statistical analyses were performed using one-way analysis of variance (ANOVA). Differences were considered significant if *p* < 0.05.

## Figures and Tables

**Table 1:** Content of Amino Groups in AG by TNBS Assay.

Entry	Feed RATIO of EDA/Gelatin (g/g)	Amino Groups Contents (mmol g^−1^)
gelatin	0	0.4903
AG-2.4	2.4	0.7236
AG-2.8	2.8	0.8438
AG-4.2	4.2	0.7851
AG-5.6	5.6	0.8073

## Data Availability

Not applicable.
